# Metasurface electrode light emitting diodes with planar light control

**DOI:** 10.1038/s41598-017-15254-3

**Published:** 2017-11-07

**Authors:** Yeonsang Park, Jineun Kim, Kyung-Sang Cho, Hyochul Kim, Min-kyung Lee, Jae-soong Lee, Un Jeong Kim, Sung Woo Hwang, Mark L. Brongersma, Young-Geun Roh, Q-Han Park

**Affiliations:** 1 Samsung Advanced Institute of Technology, 130 Samsung-ro, Yeongtong-gu, Suwon-si, Gyeonggi-do, 16678 Korea; 2 Geballe Laboratory for Advanced Materials, Stanford University, 476 Lomita Mall, Stanford, California, 94305 USA; 3 Department of Physics, Korea University, 145 Anam-ro, Seongbuk-gu, Seoul, 02841 Korea

## Abstract

The ability of metasurfaces to manipulate light at the subwavelength scale offers unprecedented functionalities for passive and active lasing devices. However, applications of metasurfaces to optical devices are rare due to fabrication difficulties. Here, we present quantum dot light emitting diodes (QDLEDs) with a metasurface-integrated metal electrode and demonstrate microscopically controlled LED emission. By incorporating slot-groove antennas into the metal electrode, we show that LED emission from randomly polarized QD sources can be polarized and directed at will. Utilizing the relation between polarization and emission direction, we also demonstrate microscopic LED beam splitting through the selective choice of polarization.

## Introduction

Planar arrays of subwavelength structures, termed metasurfaces, have been attracting interest for controlling electromagnetic waves within a subwavelength-thickness interface^1–6^. Passive-type metasurface devices such as reflectarrays or transmitarrays have been widely used to receive and transmit signals in the radio frequency (RF) and terahertz (THz) range^7–10^. More recently, metasurfaces with spatially inhomogeneous scatterers have been proposed to shape wavefronts in a desired manner^3,11^. Integration of metasurfaces with active devices has been used to control the emission and nonlinear response of quantum cascade lasers in the mid-infrared or THz regime^12–15^. It is highly desirable to adapt metasurfaces to optical frequencies as they raise the possibility of new concept planar photonics^16–20^. In particular, integrating metasurfaces with active optical devices such as light emitting diodes (LEDs) would provide unprecedented submicron-scale control over the light-matter interaction in the device to achieve improved performance or new functionalities. However, metasurface-integrated active optical devices are rare because the small size of optical scatterers causes not only fabrication difficulties but also low coupling efficiency with light emitters such as quantum wells (QWs), quantum dots (QDs), and molecule dyes.

Here, we report the first time fabrication of a metasurface-integrated LED that controls the polarization and direction of emission. We engraved a nanoscale slot-groove-array structure on the top electrode of a LED. The top electrode is made of multilayer metals for the efficient operation of the slot-groove as an optical antenna. We show that the metasurface-integrated electrode not only actuates the LED, but also steers the emission from randomly polarized sources into a linearly polarized and directed beam through polarization filtered by slot-antenna. The overall power extracted from the LED through the metasurface electrode is rather small. For example, LEDs with slot-groove-array structure of 400 nm spacing show the emission efficiency of about 4.5% compared to the electrode-free open space emission. Nevertheless, when we restrict to the opening area of holes, the area-normalized emission efficiency of the 400 nm subwavelength spacing case is about 82%^21^. We also introduced pixelated slot-groove arrays where each pixel controls the beam separately and demonstrated polarization-dependent beamsplitting through the selective choice of polarization by a detecting polarizer.

## Results and Discussion

**Figure 1:**
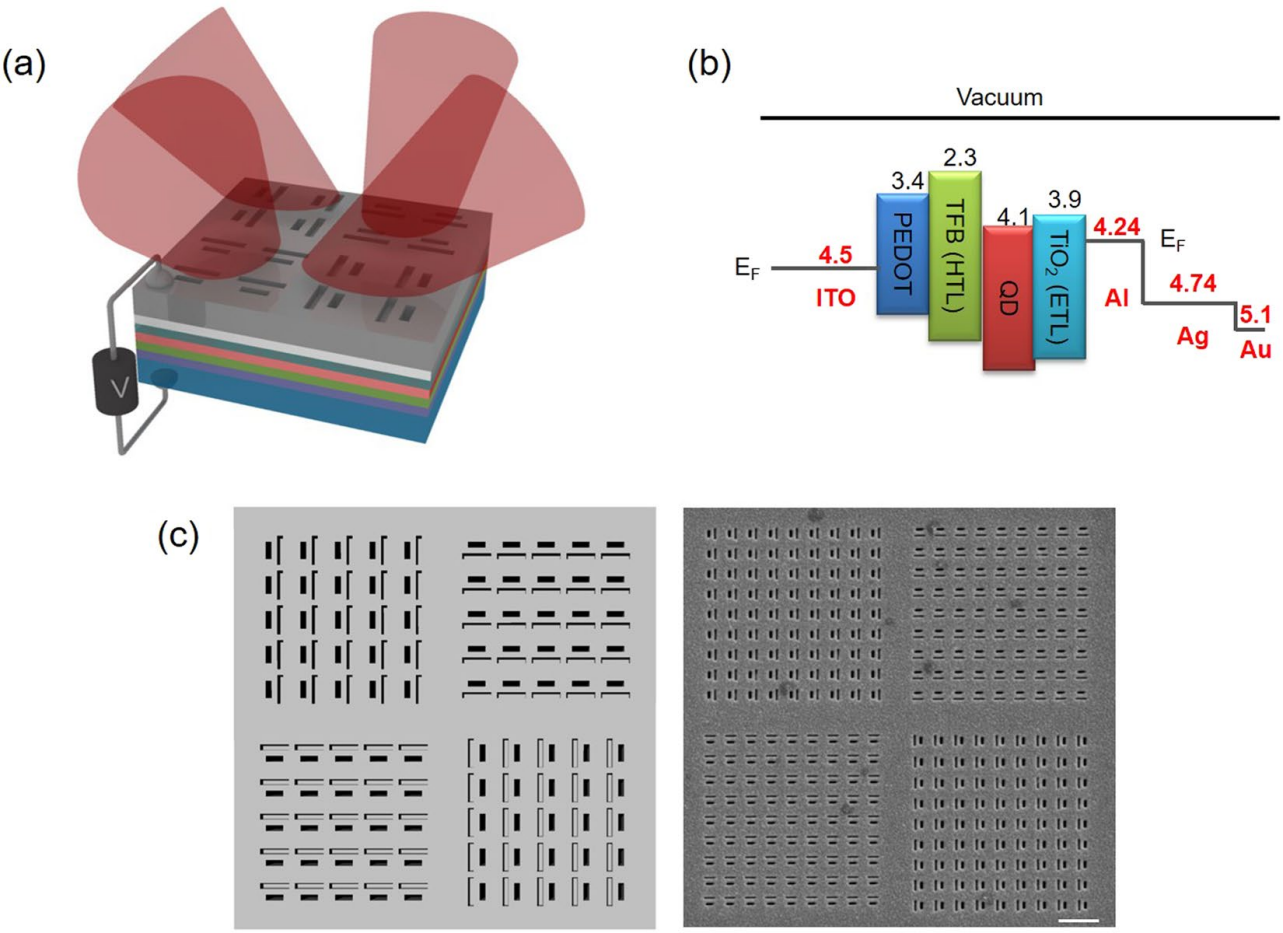
Light emitting diode with metasurface electrode. (**a**) Schematic of the colloidal quantum dot light emitting diode (CQD LED) with metasurface-integrated electrode. (**b**) Band diagram of the CQD LED. E_F_ is the Fermi energy level, HTL is a hole transport layer and ETL is an electron transport layer. Each number corresponds to the work function of the material. (**c**) Left: schematic illustration of device with pixelated four directional metasurfaces in top view. Right: Scanning electron microscope (SEM) images of fabricated device in top view. The white bar corresponds to 1000 nm.

Figure 1a shows the schematics of the LED with a metasurface-integrated electrode. The LED has a bottom p-contact electrode made of indium tin oxide (ITO) and a top n-contact metal electrode. As a light-emitting material, we used chemically-synthesized CQDs of core-multishell structure with an emission peak at 604 nm and a full-width half-maximum (FWHM) of ~33 nm^22,23^. For the electrical operation of the QD LED, we spin-coated a hole transport layer (HTL) and an electron transport layer (ETL) that sandwich the QD layer of 40 nm thickness. For the top electrode of the LED, we deposited Al, Ag, and Au metal in sequence using an electron beam evaporator. Generally, a QD LED uses Al metal alone for the top electrode because of its low work function of about 4.24 eV compared to 4.5 eV of the ITO used for the bottom electrode^24–26^. However, Al metal is highly absorbing in the visible range^24^ and not efficient for the operation of slot optical antennas. On the other hand, Ag metal has a low imaginary part of the permittivity in the visible and NIR region^24^ that is suitable for the antenna operation, but its work function is about 4.74 eV, larger than that of the ITO bottom electrode^25^, which is inefficient for carrier injection between the electrodes of the LED. These difficulties can be avoided and the superior properties of Al and Ag can be selectively utilized if we replace the top Al electrode of the QD LED in the previous report^26^ by an Al-Ag-Au multilayer metal electrode. We sequentially deposited 50 nm Al, 240 nm Ag, and 10 nm Au for the top contact electrode (Fig. 1b) where the last Au layer is a capping layer to protect the Ag from oxidation. Successful operation of the QD LED with the multilayer metal electrode was demonstrated by measuring the representative current-voltage curve of the LED^27,28^. (see Supplementary Information S1)

**Figure 2:**
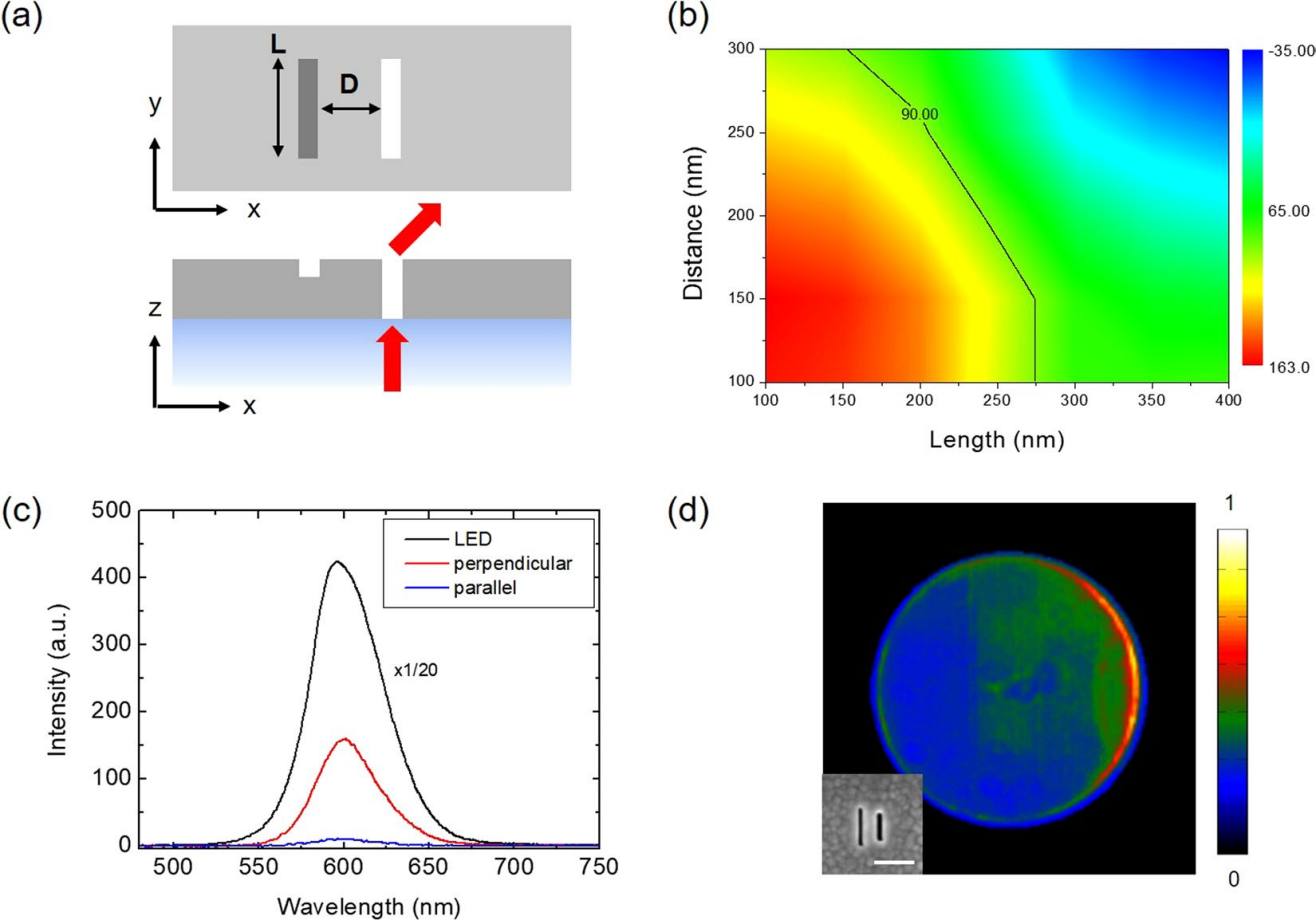
Polarized and directional radiation from the slot-groove unit. (**a**) Schematic top (top) and side view (bottom) of slot-groove unit structure. L (D) denotes length (distance) of groove structure (incident light shown as red arrow). (**b**) Contour map of phase difference between slot and groove. The black line corresponds to a phase of π/2. (**c**) Measured electroluminescence (EL) spectrum of LED. The red (blue) spectrum is measured with x- (y-) polarizer. The black line is an EL spectrum of the conventional LED. (**d**) Measured Fourier-space image of LED with slot-groove for rightward deflection. The EL intensity in Fourier-space image was normalized by the maximum intensity. The white bar in the SEM image corresponds to 200 nm.

An example of the metasurface structure on the top electrode is shown in Fig. 1c. Slot-groove arrays with 500 nm spacing are engraved, which are divided into four different pixel regions designed for the left-, up-, right-, and down-ward emissions of the LED. The slot-groove structure is particularly well adapted as an optical antenna for our LED application. Commonly used metasurface antennas, including the V-shape^3^, H-shape^29^, and arrayed antennas with gradient length^30^ or other geometrical parameters^31,32^, are of the rod-type and work under the excitation of coherent electromagnetic waves. However, the emission from QD light sources is incoherent and randomly polarized so that antenna operation such as directional beaming based on phase control is difficult to achieve. Moreover, it is difficult to control LED emission by rod-type optical antennas because the signals are easily spoiled by uncoupled light. We overcome these difficulties by introducing a new slot-groove antenna fabricated on the top electrode as shown in Fig. 1c. As an aperture-type antenna, the slot antenna is relatively easy to fabricate and controls light with high efficiency because emission from uncoupled LED sources is blocked by the metal plane. Each slot, when resonantly excited, acts efficiently as a magnetic dipole emitter with a definite phase and polarization directed perpendicularly to the long side of the slot^33^. Next to a slot, we add a groove as illustrated in Fig. 2a to form the slot-groove antenna unit. Groove structure is introduced to achieve coherent light superposition from randomly phased QD light sources and steer the emission from a slot. Emitted light from a slot interferes with the secondary light from the groove that is excited plasmonically by the slot and scattered at the groove. These two emission pathways have a definite phase relationship despite the random phases of QD sources. They interfere destructively if the phase difference is π/2 thereby deflecting the radiation towards a direction opposite to the groove. We designed the slot-groove antenna in a multilayer metal film of 300 nm thickness with 100 nm groove depth and 180 nm slot length. The slot length was determined from the single-slot transmission spectrum in order to tune resonance at the red LED wavelength. (see Supplementary Information S2) The groove size and the distance from the slot are chosen to achieve a phase difference of π/2. Figure 2b shows the contour map of the phase difference calculated numerically using the finite-difference time-domain (FDTD) method. (see Supplementary Information S3) Phase differences are calculated varying the distance from 100 nm to 300 nm by 50 nm, and the groove length from 100 nm to 400 nm by 50 nm. We designed the slot-groove structure based on the contour map with 180 nm slot length, 280 nm groove length, 150 nm separation distance, and fabricated the slot-groove array on the top metal electrode of the LED by focused ion beam (FIB) milling method.

**Figure 3:**
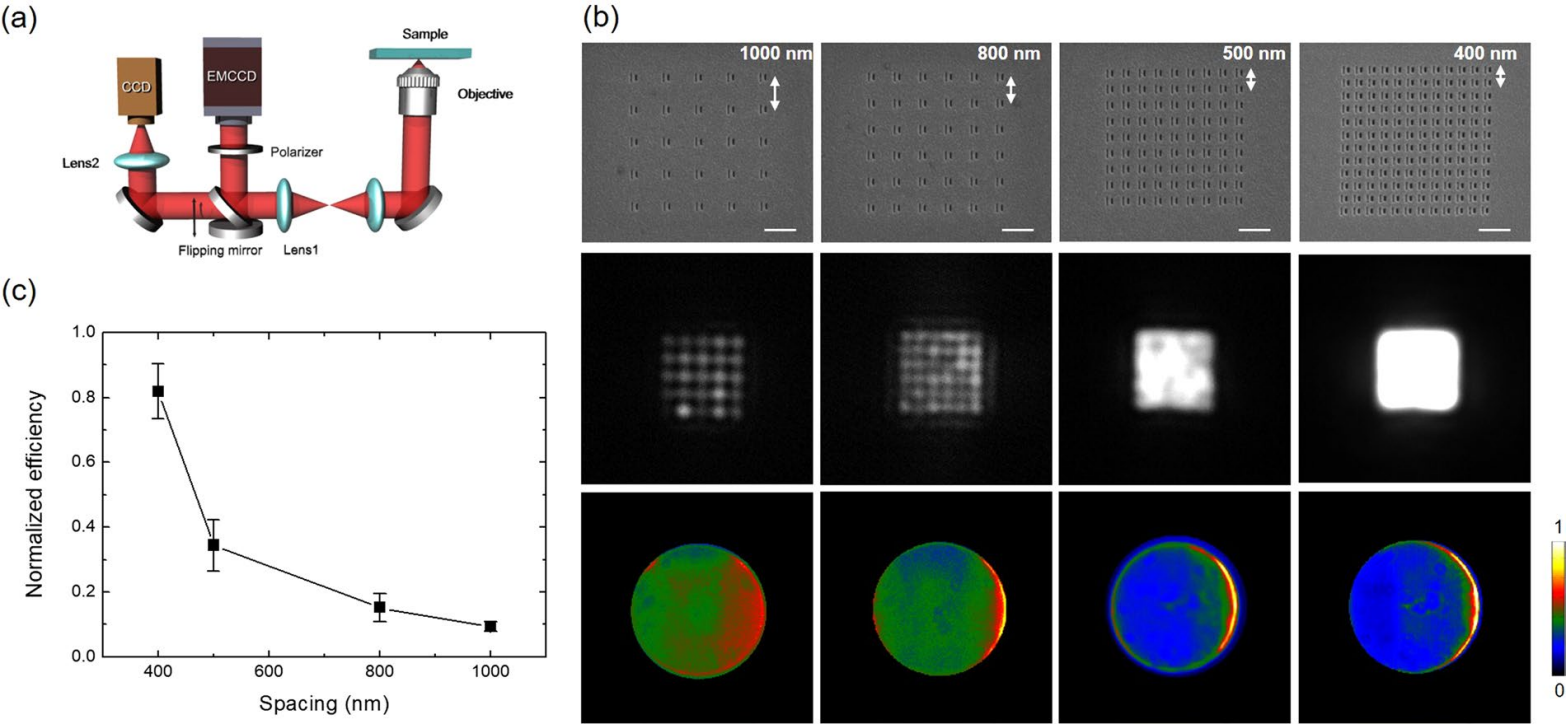
Intensity of LED emission with different spacing of slot-groove elements. (**a**) Schematics of optical and Fourier-space image measurement setup. (**b**) The SEM images correspond to fabricated slot-groove array samples with 1000 nm, 800 nm, 500 nm, and 400 nm spacing. The white bar in the SEM image corresponds to 1000 nm. Measured optical and Fourier-space images of EL emission are shown in second and third row respectively. The scale bar in Fourier-space images shows the intensity normalized by its maximum. (**c**) Slot-area-normalized intensity of each sample with different spacing.

**Figure 4:**
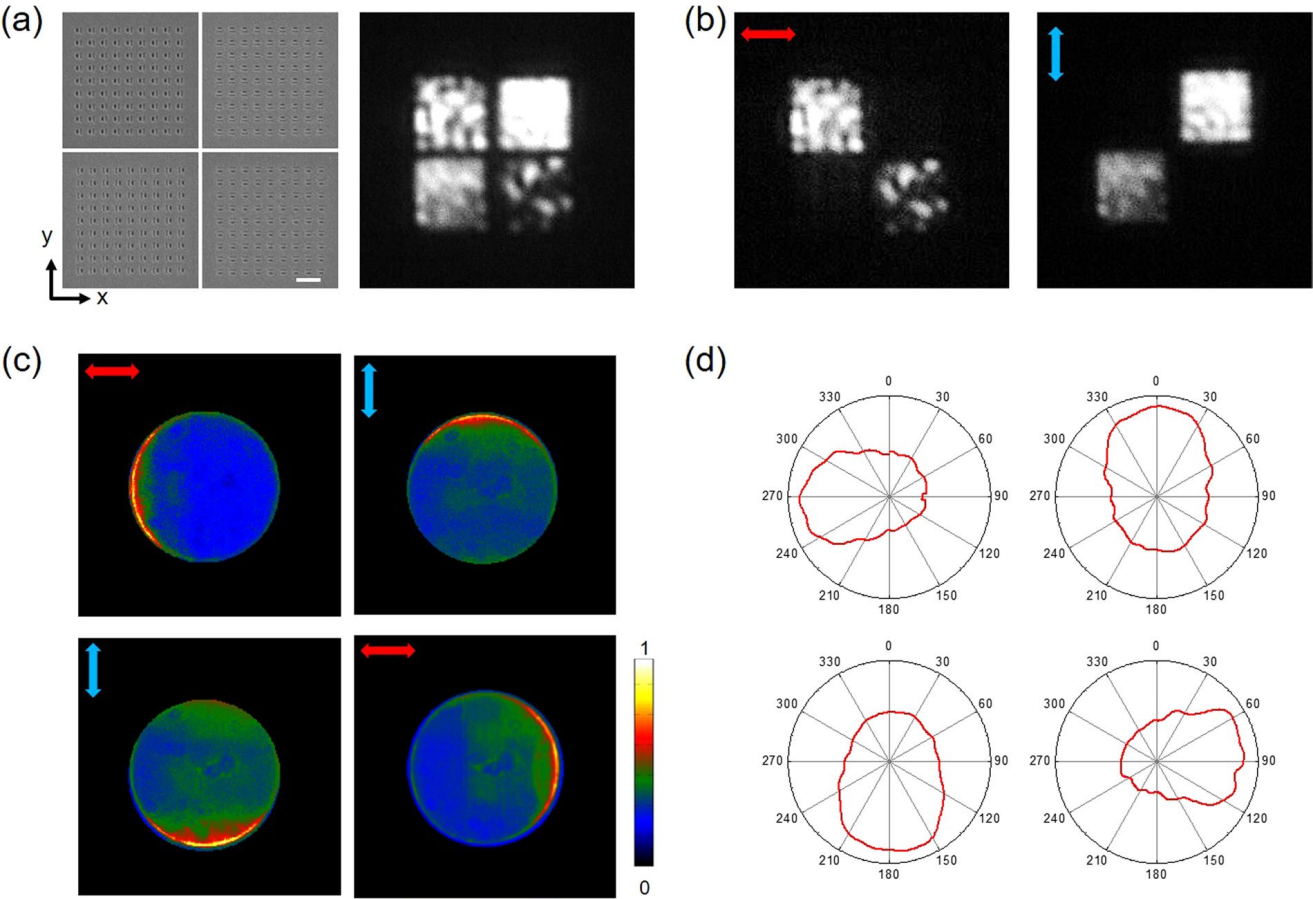
Polarized directional emission from metasurface electrode light emitting diode. (**a**) SEM images of 4-directional slot-groove unit (Left) and measured optical image of EL from LED with pixelated metasurface for 4-directional emission. (Right) The white bar in SEM images corresponds to 1000 nm. The optical image was measured without a polarizer. (**b**) Measured optical images with a directional polarizer. Red (blue) arrow shows the direction of x- (y-) polarizer in each optical image. (**c**) Measured Fourier-space images of EL from LED with pixelated metasurface for 4-directional emission. The scale bar shows the normalized intensity. (**d**) ϕ-polar plots of measured Fourier-space images.

To test our metasurface LED, we measured the electroluminescence (EL) spectrum, the real-space (optical) and Fourier-space image of emitted light. We used the measurement setup shown in Fig. 3a to obtain optical and Fourier-space images simultaneously. The polarization-resolved EL spectra taken from a LED with a slot-groove antenna are given in Fig. 2c. Unlike the unpolarized emission of a conventional LED (black curve), antenna-controlled emission is strongly polarized (red curve) perpendicularly to the long side of slot and displays a nearly identical spectral peak around 604 nm. Figure 2d is the Fourier-space image of LED emission through a slot-groove antenna, which clearly shows the deflection of emitted light toward the direction opposite to the groove. From the Fourier-space image, we find the deflection angle to be ~35°. (see Supplementary Information S4). The functionality of metasurfaces patterned on the electrode has been investigated varying the slot-groove array spacing by 1000 nm, 800 nm, 500 nm, and 400 nm as shown in Fig. 3. We find that LED emission increases for smaller spacing in accordance with the increased number of slots while the directional control of emission is maintained for all four cases as shown in Fig. 3b. Notably, the 400 nm subwavelength spacing shows about 82% emission efficiency in comparison with the hole-area-normalized intensity. (see Supplementary Information S5) This confirms that slot-groove array with subwavelength spacing indeed works as a metasurface electrode. To demonstrate the capability of controlling the emission direction, we fabricated the structure for multi-directional emission with slot-groove arrays in four different pixel regions and measured the emission with the results given in Fig. 4. The spacing of slot-groove elements is fixed as 500 nm based on the above result shown in Fig. 3 and the pixels are separated by 1 μm. Figure 4a is a scanning electron microscope (SEM) image of the fabricated slot-groove unit and an optical image of the EL. The optical images of Fig. 4b measured with polarizers clearly show the polarization properties of metasurface LED emission. Figure 4c shows Fourier-space images corresponding to each pixel. Polar plots of emission are obtained from the Fourier-space images and presented in Fig. 4d. These results clearly show that emitted light from the metasurface LED in Fig. 4a is deflected in four distinct directions.

**Figure 5:**
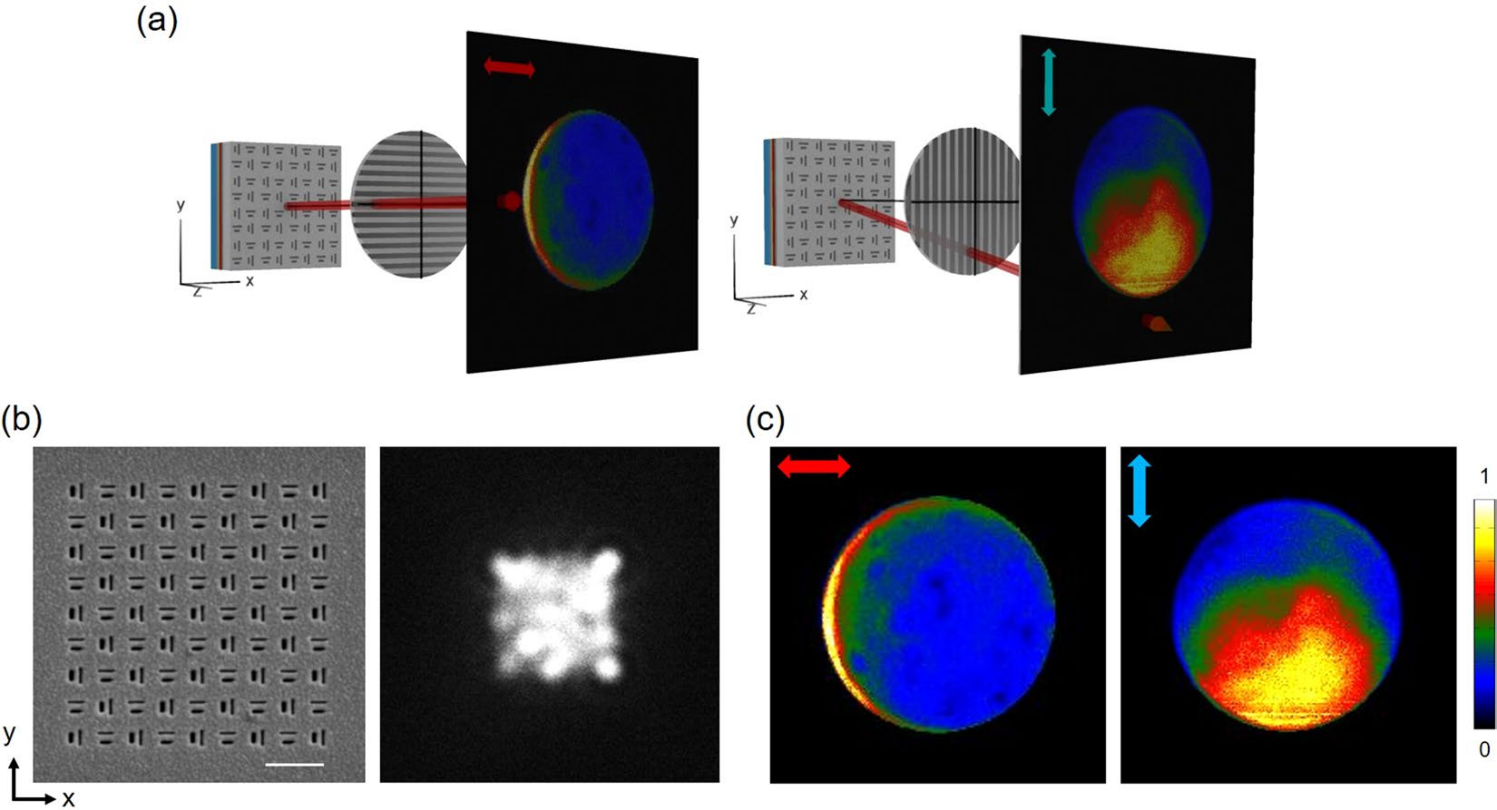
LED beam splitting by selective polarization. (**a**) Schematics of polarization beam splitter using metasurface. (**b**) SEM images of slot-groove arrays with 500 nm spacing for two-orthogonal directions. Measured optical image without a polarizer is shown in right figure. The white line in SEM image corresponds to 1000 nm. (**c**) By changing the direction of polarizer from x-axis to y-axis, the direction of LED emission is changed from left-wards to down-wards. The scale bar shows the normalized intensity.

Since each slot-groove antenna can steer light independently, the metasurface LED can generate locally varying and microscopically controlled light. Figure 5 is one example of a microscopically designed light source with alternating antenna directions. This results in a light source with alternating polarization and direction at the wavelength scale as shown in Fig. 5b. By applying two orthogonal polarizers selectively as illustrated in Fig. 5a, we confirmed that the LED emissions consist of two distinct and orthogonally polarized emissions into different directions from the measured Fourier-space images shown in Fig. 5c.

## Conclusion

In conclusion, we have fabricated an LED with a metasurface-integrated electrode and demonstrated microscopically controlled polarization and directional emissions in the visible regime. The multi-functional metasurface can be utilized to design light sources with arbitrarily varying local polarization and direction because each unit element of the metasurface electrode can steer light independently by changing its polarization and direction. Thus, we expect that metasurface-integrated LEDs will be useful for the 3D display panels, holography, and nanoscale integrated photonic chips.

## Methods

### Fabrication of colloidal quantum dot light emitting diode

The colloidal quantum dots (CQDs) of CdSe/CdS/ZnS core-shell-shell were synthesized by the process reported in previous publications^21,22^. The CQDs dispersed in cyclohexane were characterized by a UV-vis spectrometer (Varian Cary 5000) and a fluorometer (Fluorolog JOBIN YVON Horiba). The wavelengths of the first excitation absorption and maximum photoluminescence (PL) were measured to be 584 nm and 604 nm, respectively. The FWHM of the CQD emission was ~ 33 nm. ITO-deposited glass was used as a substrate for the CQD LED fabrication. This ITO layer was patterned by chemical wet etching. Poly(3,4-ethylenedioxythiophene)-poly(stryenesulfonate) (PEDOT-PSS) and poly(9,9-dioctyl-fluorene-co-N-(4- butylphenyl)diphenylamine) (TFB) were spin-coated as the HTL for hole injection. The synthesized CQDs were spin-coated at 2000 rpm to match the thickness of 40 nm for efficient carrier transport. After that, gel-type TiO_2_ was spin-coated as the ETL for electron injection. As the final process, Al, Ag, and Au metals with 50 nm, 240 nm, and 10 nm thicknesses, respectively, were sequentially deposited by electron beam evaporation. Metasurfaces made of slot-grooves were patterned by a FIB milling machine (FEI Helios NanoLab) on the top metal electrode of the fabricated LED.

The fabricated LED was characterized in an IVL measurement tester (Polaronix M6100, McScience). The electrode of the LED was connected by copper wire and voltage was supplied in steps of 1 V by a source meter (Keithley 2400, Keithley Instrument Inc.). The current-voltage relation of the LED and the EL spectrum were simultaneously measured.

### Simulation

FDTD simulations were executed in 3-dimensional (3D) model using an FDTD simulator (ver.8.15.7) of Lumerical Solutions. The optical constants in ref.^34^ were used in 3D simulation. The far-field radiation pattern of 3D model was calculated by the aid of near-to-far-field transformation analysis method^35^.

### Measurement

The optical measurement of the sample was executed on an inverted microscope (Olympus IX81). Emission from the LED with metasurface-integrated electrode was collected by a 100 × microscope objective (Olympus UPlanFL N, NA = 0.95) and optical images were obtained by an SCMOS image sensor (INFINITY 3, Numenera Inc.). The collected emission was directed to a spectrometer with 350 μm entrance slit width (Spectro Pro 500i, Action Research Inc.) and detected by an electron-multiplying charge-coupled device (EMCCD) (Newton^EM^, ANDOR).

The Fourier-space images^36,37^ were measured using the setup shown in Fig. 3a. To obtain the Fourier-space image, a convex lens was placed at the image plane of the microscope, and then an EMCCD detector was positioned at the focal plane of the lens (*f*). The image taken from the EMCCD is a Fourier-space image. By switching the mirror positioned in the light path, we could easily change the light path from the microscope. To convert the Fourier-space image into an optical image, we positioned another lens with the same *f* at the distance of 2 *f* from the first lens. The transmitted light was focused into another CCD device by the second lens, and the optical image was obtained in this CCD.

## Electronic supplementary material

supplementary information

## References

[1] Kildishev AV, Boltasseva A, Shalaev VM (2013). Planar photonics with metasurfaces. Science.

[2] Yu N, Capasso F (2014). Flat optics with designer metasurfaces. Nat. Mater..

[3] Yu N (2011). Light propagation with phase discontinuities: generalized laws of reflection and refraction. Science.

[4] Li X (2012). Flat metasurfaces to focus electromagnetic waves in reflection geometry. Opt. Lett..

[5] Arbabi A, Horie Y, Bagheri M, Faraon A (2015). Dielectric metasurfaces for complete control of phase and polarization with subwavelength spatial resolution and high transmission. Nat. Nano..

[6] Yu YF (2015). High‐transmission dielectric metasurface with 2π phase control at visible wavelengths. Laser & Photonics Reviews.

[7] Pozar DM, Targonski SD, Syrigos H (1997). Design of millimeter wave microstrip reflectarrays. IEEE Trans. Antennas. Propag..

[8] Kuester EF, Mohamed M, Piket-May M, Holloway CL (2003). Averaged transition conditions for electromagnetic fields at a metafilm. IEEE Trans. Antennas. Propag..

[9] Holloway CL (2009). A discussion on the interpretation and characterization of metafilms/metasurfaces: The two-dimensional equivalent of metamaterials. Metamaterials.

[10] Ryan CG (2010). A wideband transmitarray using dual-resonant double square rings. IEEE Trans. Antennas. Propag..

[11] Ni X, Emani NK, Kildishev AV, Boltasseva A, Shalaev VM (2012). Broadband light bending with plasmonic nanoantennas. Science.

[12] Yu N (2008). Small-divergence semiconductor lasers by plasmonic collimation. Nat. Photon..

[13] Yu N, Wang Q, Capasso F (2012). Beam engineering of quantum cascade lasers. Laser & Photonics Reviews.

[14] Rauter P (2014). Electrically pumped semiconductor laser with monolithic control of circular polarization. Proc. Natl. Acad. Sci..

[15] Lee J (2014). Giant nonlinear response from plasmonic metasurfaces coupled to intersubband transitions. Nature.

[16] Ni, X., Kildishev, A. V. & Shalaev, V. M. Metasurface holograms for visible light. *Nat. Commun*. **4** (2013).

[17] Huang, L. *et al*. Three-dimensional optical holography using a plasmonic metasurface. *Nat. Commun*. **4** (2013).

[18] Li Z, Palacios E, Butun S, Aydin K (2015). Visible-frequency metasurfaces for broadband anomalous reflection and high-efficiency spectrum splitting. Nano Lett..

[19] Esfandyarpour M, Garnett EC, Cui Y, McGehee MD, Brongersma ML (2014). Metamaterial mirrors in optoelectronic devices. Nat. Nanotechnol..

[20] Zheng G (2015). Metasurface holograms reaching 80% efficiency. Nat. Nanotechnol..

[21] Kyoung JS (2010). Far field detection of terahertz near field enhancement of sub-wavelength slits using Kirchhoff integral formalism. Opt. Commun..

[22] Lim J (2007). Preparation of highly luminescent nanocrystals and their application to light‐emitting diodes. Adv. Mat..

[23] Cho KS (2009). High-performance crosslinked colloidal quantum-dot light-emitting diodes. Nat. Photon..

[24] Michaelson HB (1977). The work function of the elements and its periodicity. J. Appl. Phys..

[25] Park Y, Choong V, Gao Y, Hsieh B, Tang C (1996). Work function of indium tin oxide transparent conductor measured by photoelectron spectroscopy. Appl. Phys. Lett..

[26] Dabbousi B, Bawendi M, Onitsuka O, Rubner M (1995). Electroluminescence from CdSe quantum‐dot/polymer composites. Appl. Phys. Lett..

[27] Colvin V, Schlamp M, Alivisatos A (1994). Light-emitting diodes made from cadmium selenide nanocrystals and a semiconducting polymer. Nature.

[28] Schlamp M, Peng X, Alivisatos A (1997). Improved efficiencies in light emitting diodes made with CdSe (CdS) core/shell type nanocrystals and a semiconducting polymer. J. Appl. Phys..

[29] Mandal P, Ramakrishna SA, Patil R, Gopal AV (2013). Polarization dependent color switching by extra-ordinary transmission in H-slit plasmonic metasurface. J. Appl. Phys..

[30] Lin D, Fan P, Hasman E, Brongersma ML (2014). Dielectric gradient metasurface optical elements. Science.

[31] Chen X (2012). Dual-polarity plasmonic metalens for visible light. Nat. Commun..

[32] Huang L (2012). Dispersionless phase discontinuities for controlling light propagation. Nano Lett..

[33] Kim J (2014). Babinet-inverted optical Yagi–Uda antenna for unidirectional radiation to free space. Nano Lett..

[34] Palik, E. D. *Handbook of optical constants of solids* (Academic Press, 1998).

[35] Taflove, A. & Hagness, S. C. *Computational Electrodynamics: The Finite-difference Time-domain Method* (Artech House, 2005).

[36] Teich, M. C. & Saleh, B. E. *Fundamentals of Photonics* (Wiley, 1991).

[37] Curto AG (2010). Unidirectional emission of a quantum dot coupled to a nanoantenna. Science.

