# Supplementary material for: Metasurface electrode light emitting diodes with planar light control
Source: Sci Rep. 2017 Nov 7;7:14753. doi: 10.1038/s41598-017-15254-3 (PMC5677004; doi:10.1038/s41598-017-15254-3)
Supplement: Supplementary file 1 — supplementary information [file 41598_2017_15254_MOESM1_ESM.doc]

**Supplementary Information**

**Metasurface electrode light emitting diodes with planar light control**

Yeonsang Park^1^*, Jineun Kim^1*^, Kyung-Sang Cho^1^, Hyochul Kim^1^, Min-kyung Lee^1^, Jae-soong Lee^1^, Un Jeong Kim^1^, Sung Woo Hwang^1^, Mark L. Brongersma^2^, Young-Geun Roh^1^†, and Q-Han Park^3^†

*^1^Samsung Advanced Institute of Technology, 130 Samsung-ro, Yeongtong-gu, Suwon-si, Gyeonggi-do, 16678, Korea.*

*^2^Geballe Laboratory for Advanced Materials, Stanford University, 476 Lomita Mall, Stanford, California 94305, USA*

*^3^Department of Physics, Korea University, 145 Anam-ro, Seongbuk-gu, Seoul, 02841, Korea*

*These authors contributed equally to this work.

†Correspondence: YG Roh, Email: [yg000.roh@samsung.com](mailto:yg000.roh@samsung.com); QH Park, Email: [qpark@korea.ac.kr](mailto:qpark@korea.ac.kr)

Table of Contents

[S1. Characterization of a quantum dot light emitting diode 2](#__RefHeading___Toc497253426)

[S2. Linear relation between resonant wavelength and length of slot antenna 2](#__RefHeading___Toc497253427)

[S3. Design of groove by 3D FDTD simulation 3](#__RefHeading___Toc497253428)

[S3-1. Calculation of electric field 3](#__RefHeading___Toc497253429)

[S3-2. Phase difference contour map 4](#__RefHeading___Toc497253430)

[S3-3. Distribution of far-field radiation patterns 5](#__RefHeading___Toc497253431)

[S4. Analyzing the deflection angle 5](#__RefHeading___Toc497253432)

[S5. Calculation of the emission efficiency 6](#__RefHeading___Toc497253433)

[References 8](#__RefHeading___Toc497253434)

[Figures and Figure captions 9](#__RefHeading___Toc497253435)

[Table and Table Caption 15](#__RefHeading___Toc497253436)

**S1. Characterization of a quantum dot light emitting diode**

We characterized the electrical and optical properties of the fabricated light emitting diode (LED) using an IVL tester. The LED was operated by supplying voltage in 1 V increments. The IVL curves are presented in Figure S1a. It displayed a similar characteristic LED curve to that previously reported^1,2^. The LED turn-on voltage was measured as ~3 V. Figure S1b shows electroluminescence (EL) spectra measured at each applied voltage. We deduced from this measurement that the peak wavelength and full-width at half maximum (FWHM) of LED emission was ~604 nm and 33 nm, respectively. The peak wavelength and FWHM were obtained by fitting the spectrum as a Gaussian form. To check the stability of the metasurface-integrated LED, all data were measured twice, before and after focused ion beam (FIB) milling fabrication of the metasurface on the top electrode, with no change detectable. All data shown in the maintext were measured at an applied voltage of 8 V because the peak wavelength of LED emission was red-shifted a little at applied voltages higher than 8 V.

**S2. Linear relation between resonant wavelength and length of slot antenna**

Because the resonant wavelength of a slot antenna increases linearly with increasing antenna length^3,4^, we should deduce the optimal length of an antenna for 604 nm peak wavelength LED emission. We executed a transmission spectrum measurement using a single slot antenna. We fabricated a slot antenna on Al-Ag-Au metal film with a total thickness of 300 nm and changed its length by 20 nm steps from 120 nm to 200 nm using a focused ion beam (FIB) milling method. (scanning electron microscope (SEM) images of Figure S2a) From the measured transmission spectra shown in Figure S2b, we obtained the linear relation *λ (nm) =* 1.37 *l(nm) +* 353.37 where *λ *is the resonant peak wavelength of the antenna and *l* is the antenna length^4^. (Figure S2c) To confirm the obtained results, we calculated the spectra of the slot antenna as a function of its length using 3-dimensional finite-difference time-domain (3D FDTD) simulation. From the spectra shown in Figure S2b and S2d, we could see that the simulated results agreed well with the measured results. The simulated linear relation between the resonant wavelength and antenna length was *λ (nm) =* 1.89 *l(nm) +* 295.14. According to the linear relation obtained from the measured transmission spectra, we chose 180 nm as the antenna length resonant with the 604 nm peak wavelength of the LED emission.

**S3. Design of groove by 3D FDTD simulation**

**S3-1. Calculation of electric field**

As mentioned in the maintext, the phase difference between the slot and groove structure provides important information about the interference and radiation direction. According to the slot antenna theory, the electromagnetic field and phase of a slot antenna are determined by the magnetic dipole current where $\vec{J}_{ms}$ is the magnetic dipole current at the opening of the slot, is the normal unit vector on the surface, and $\vec{E}_{t}$ is the tangential electric field (E-field) over the slot structure^5^. Since it is well-known that an optical slot antenna has linear-polarization perpendicular to its length (x-polarization)^6,7^, the y- and z-components of E-field are very weak compared to the x-component of the E-field. Finally, the magnetic dipole current of a slot antenna is directly proportional to *E_x_*. Figure S3a shows the calculated *E_x_*-field of the proposed structure in Figure 2b of the maintext. We fixed the length of the slot antenna as 180 nm. While changing the length of groove (L) from 100 nm to 400 nm by 50 nm and the distance between slots and grooves (D) from 100 nm to 300 nm by 50 nm, we executed a 3D FDTD simulation. The wavelength of incident plane light was 604 nm, which was the peak wavelength of LED emission. The *E_x_*-field distributions calculated in all cases were drawn in matrix form in Figure S3a. We could see that a strong *E_x_*-field mode was formed inside the slot antenna and was almost uniform in all cases regardless of its finite dimensions and existence of the groove structure. Therefore, we can define a single-valued phase by taking the phase of *E_x_* at the center of the slot antenna. The uniformity of the phase inside the slot antenna implies that the slot antenna can be regarded as a single magnetic dipole source. The phase of the groove structure can be also calculated from the *E_x_* field in a similar manner to the slot antenna case.

When the distance (D) between the slot and groove was fixed at 100 nm, we could see that another mode was formed inside the groove structure, with the maximum field intensity at a length of 250 nm that became weaker as the groove length increased. This means that the coupling between the slot antenna and groove was strongest when the groove length was 250 nm. From the simulated far-field radiation patterns presented in Figure S3c, we could deduce that this strong coupling resulted in efficient deflection control of the slot-groove structure. To understand this coupling, we analyzed the phase difference between slots and grooves.

**S3-2. Phase difference contour map**

The phase difference between a slot and groove can be defined as the subtraction of two phases. Figure S2b shows the contour map of the phase difference calculated using 3D FDTD. All conditions were identical to the above *E_x_*-field calculation. Given a fixed distance, we could see that the phase difference decreased, and crossed the line of π/2 drawn as the black line in the contour map, as the length was increasing. Comparing the far-field distributions shown in Figure S2c, we could see that constructive interference occurred in the upper region of the contour map divided by the black line, and the transmitted light was deflected toward the position where the groove was located. On the contrary, destructive interference occurred in the lower region of the contour map and the transmitted light was deflected toward the opposite position from the groove location.

It is worth noting that adding more grooves in addition to the first groove can increase directionality of the deflected light. However, since the intensity of surface plasmons generated by the slot antenna exponentially decays while propagating and is continuously scattered by the groove structure, the directivity increment by adding elements is weak. Therefore, a simple structure made with only two elements of one slot and one groove is enough to create directional emission. This simple unit made of one slot and one groove has the advantages of easy fabrication and a small footprint for real applications of metasurfaces.

**S3-3. Distribution of far-field radiation patterns**

To understand the deflection directions clearly, we calculated the far-field radiation pattern. Using the near-to-far transformation method, we projected the calculated near-field of the structure into the upper hemisphere with a radius of 1 m, with the slot-groove structure located at the origin. The far-field radiation pattern was obtained by projecting the hemisphere into a circle. This calculated 2D image corresponded to the experimentally measured Fourier-space image. Figure S2c shows the far-field pattern distribution calculated by changing D and L of the slot-groove structure by the same conditions used in the field and phase difference calculation. From Figure S2c, we could see that the deflection angle of transmitted light changed depending on the parameters D and L.

**S4. Analyzing the deflection angle**

To deduce the deflection angle from the measured Fourier-space image, we analyzed the relation between the pixels of the 2D CCD image obtained by the Fourier-space image measurement and the deflection angle θ. First, we measured the Fourier-space image of the 2D grating with 2 μm spacing and obtained the 2D diffraction pattern shown in Figure S4a. The diffraction pattern was obtained with two lasers with 664 nm and 403 nm wavelength. The deflection angle of the *m*-th order peak can be calculated from the grating diffraction relation of *d∙sinθ = mλ* where *d* is the grating spacing, *m* is an integer, and *λ* is the incident wavelength^8^. In case of the 664 nm laser, the 1st-order (2nd-order) diffraction intensity had a deflection angle of 19.33° (41.45°). By counting the pixel position of the *m*-th order intensity, we obtained the graph shown in Figure S4c where the data from the 664 nm laser are presented as red dots and the data from the 403 nm laser as blue dots. Finally, the red and blue dots could be fitted as the function of *pixel position = R∙sinθ *where R is a fitting parameter. This fitting function could be obtained from the geometry between the 2D CCD image and the hemisphere where the sample was located at the origin, as shown in Figure S4b. The green line in the graph of Figure S4c corresponded to the fitted curve. We obtained a fitting parameter of 228.2. Using this relation between the pixel position of the CCD image and the deflection angle θ, we found the deflection angle by counting the pixel position of the highest intensity in the measured Fourier-space image of emission from the device as shown in Figure S4d.

**S5. Calculation of the emission efficiency**

In the maintext, the hole-area-normalized intensity means the slot-intensity normalized by the reference slot-intensity. When the intensity of *I_1_* through one period area (*S_1_ = p*p*) is measured, the intensity of *I_1_* can be calibrated into the intensity of *I_1_’* measured through one-slot area (*S_2_ = w*l*). Therefore, we can know that the calibrated intensity of *I_1_’* corresponds to the intensity *I_1_* multiplied by factor of *S_2_/S_1_* (one slot-area with respect to one lattice-area) from the Fig. S5. This is the reference slot-intensity. The nomenclature of the area-normalization comes from area factor of *S_2_/S_1_*^9^. Finally, the hole-area-normalized intensity (*I_area_*) becomes into the *I_2_/I_1_’*, and we call this parameter as the hole-area-normalization emission efficiency in the maintext. On the contrary, if the measured intensity of *I_2_* transmitted through one slot-area is normalized by *I_1_* transmitted through one lattice-area, we can define this normalized number as an absolute emission efficiency of slot-groove array with its period. (*I_abs_=(I_2_*#)/(I_1_**#*)=I_2_/I_1_*, where # is the number of the array*)*.

From the measured intensities of slot-groove array with the spacing of 400 nm, 500 nm, 800 nm, and 1000 nm, we calculated an absolute unit-cell and hole-area-normalized emission efficiencies, respectively. The figure S6 and table S1 show *I_abs_* and *I_area_* obtained by experiment.

**References**

1 Cho, K. S. *et al.* High-performance crosslinked colloidal quantum-dot light-emitting diodes. *Nat. Photon.* **3**, 341-345 (2009).

2 Kim, T. H. *et al.* Full-colour quantum dot displays fabricated by transfer printing. *Nat. Photon.* **5**, 176-182 (2011).

3 Garcia-Vidal, F. J., Martin-Moreno, L., Ebbesen, T. & Kuipers, L. Light passing through subwavelength apertures. *Rev. Mod. Phys.* **82**, 729 (2010).

4 Kim, J. *et al.* Babinet-inverted optical Yagi–Uda antenna for unidirectional radiation to free space. *Nano Lett.* **14**, 3072-3078 (2014).

5 Balanis, C. A. *Antenna Theory: Analysis and Design* (Wiley-Interscience, 2005).

6 Degiron, A., Lezec, H., Yamamoto, N. & Ebbesen, T. Optical transmission properties of a single subwavelength aperture in a real metal. *Opt. Commun.* **239**, 61-66 (2004).

7 Ren, M. *et al.* Linearly polarized light emission from quantum dots with plasmonic nanoantenna arrays. *Nano Lett.* **15**, 2951-2957 (2015).

8 Teich, M. C. & Saleh, B. E. *Fundamentals of Photonics* (Wiley, 1991).

9 Kyoung, J. S. *et al.* Far field detection of terahertz near field enhancement of sub-wavelength slits using Kirchhoff integral formalism. *Opt. Commun.* **283**, 4907–4910 (2010).

**Figures and Figure captions**

**
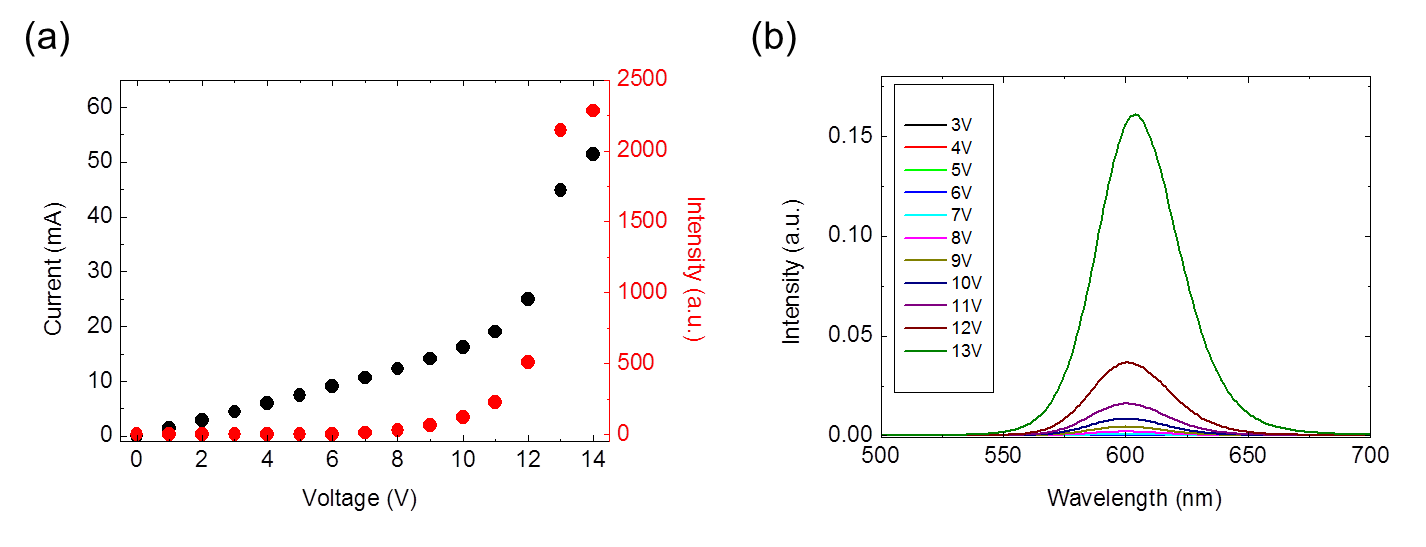
**

**Figure S1. Electrical and optical properties of LED with metasurface electrode** (a) The current of LED device and EL intensity versus applied voltage. Black and red circles correspond to the measured current and EL intensity, respectively. (b) Optical spectra of LED emission measured by changing the voltage in 1 V increments.

**
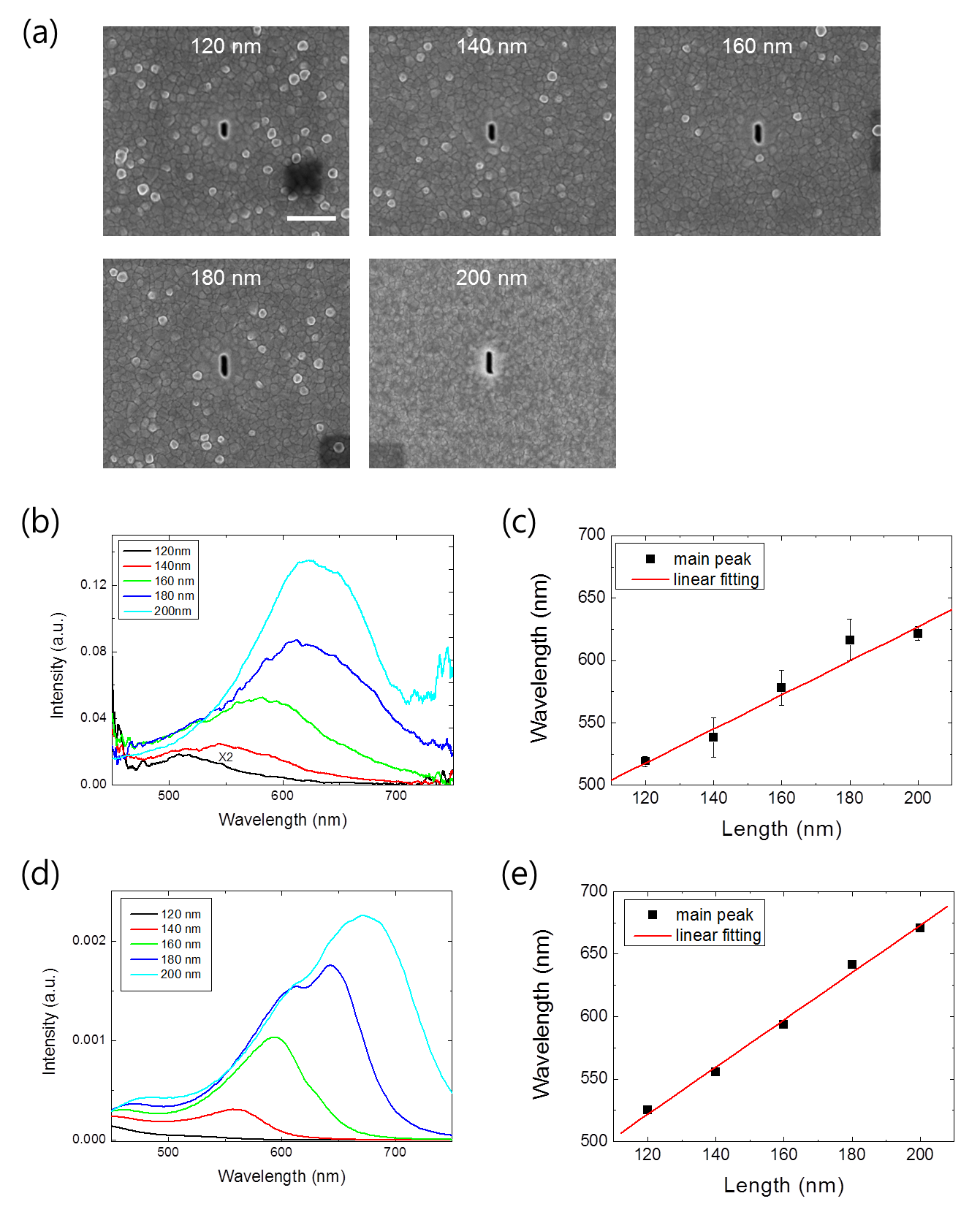
**

**Figure S2. Linear relation between resonant peak wavelength and length of single slot antenna** (a) SEM images of different lengths of the fabricated slot antenna from 120 nm to 200 nm in 20 nm increments. White line corresponds to 500 nm and all images were obtained at the same magnification. (b) The transmission spectra measured in each antenna. (c) Black rectangles show peak wavelength and are fitted linearly by a red line. (d) The transmission spectra calculated by 3D FDTD simulation. (e) Black rectangles show peaks of calculated transmission and are fitted linearly by a red line.

**
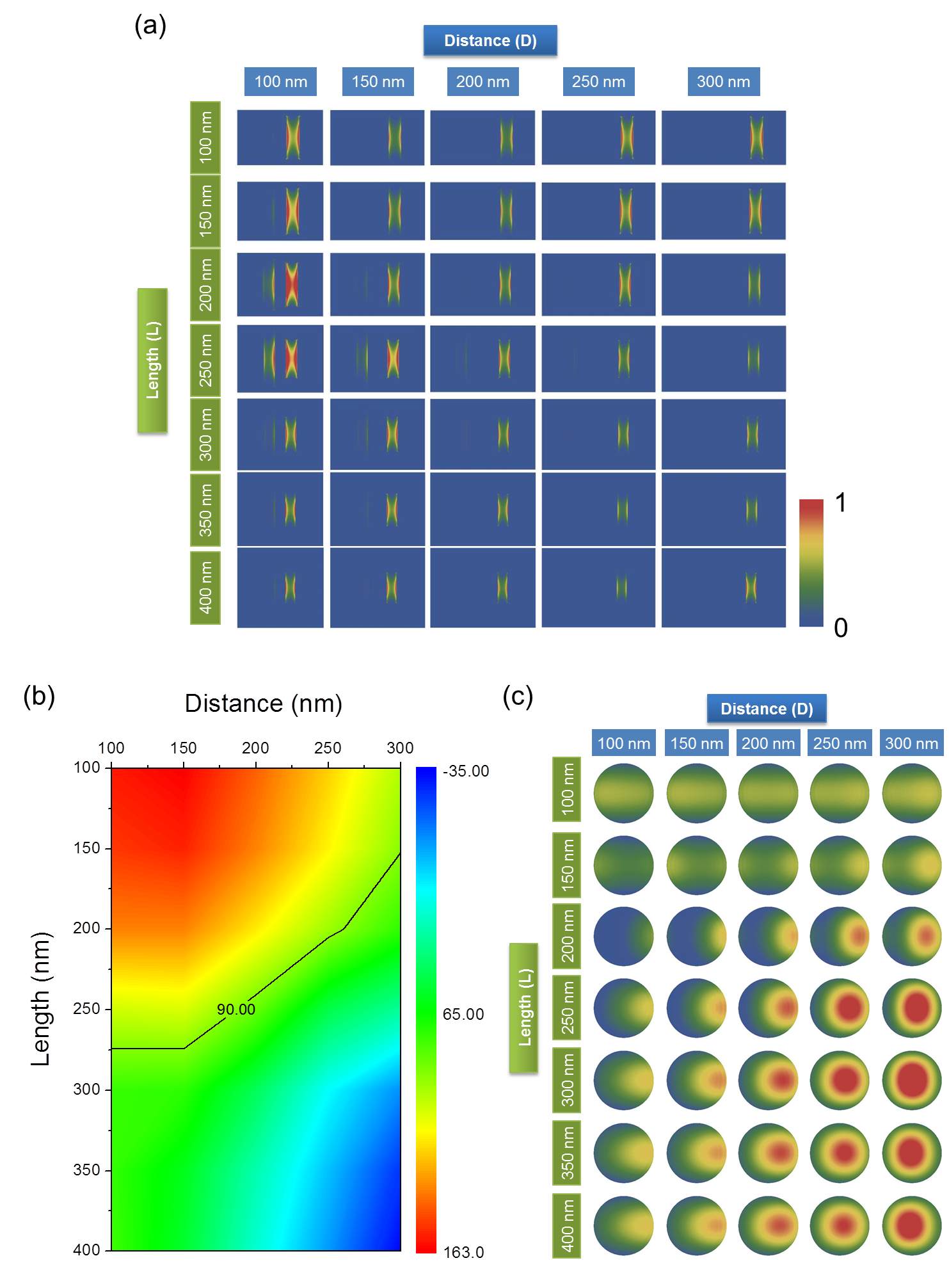
**

**Figure S3. Analysis of slot and groove structure** (a) Matrix map of the normalized *E_x_*-field intensity of slot and groove structure for changed parameters of L and D. The scale bar shows the normalized field intensity. (b) Contour map of the calculated phase difference between slot and groove for changing L and D. The black line corresponds to π/2. The number in the scale bar shows the angle of phase difference in degrees. (c) Matrix map of the normalized far-field radiation pattern. The field intensity was projected on the circle. The color map identical to scale bar in (a) is used.

**
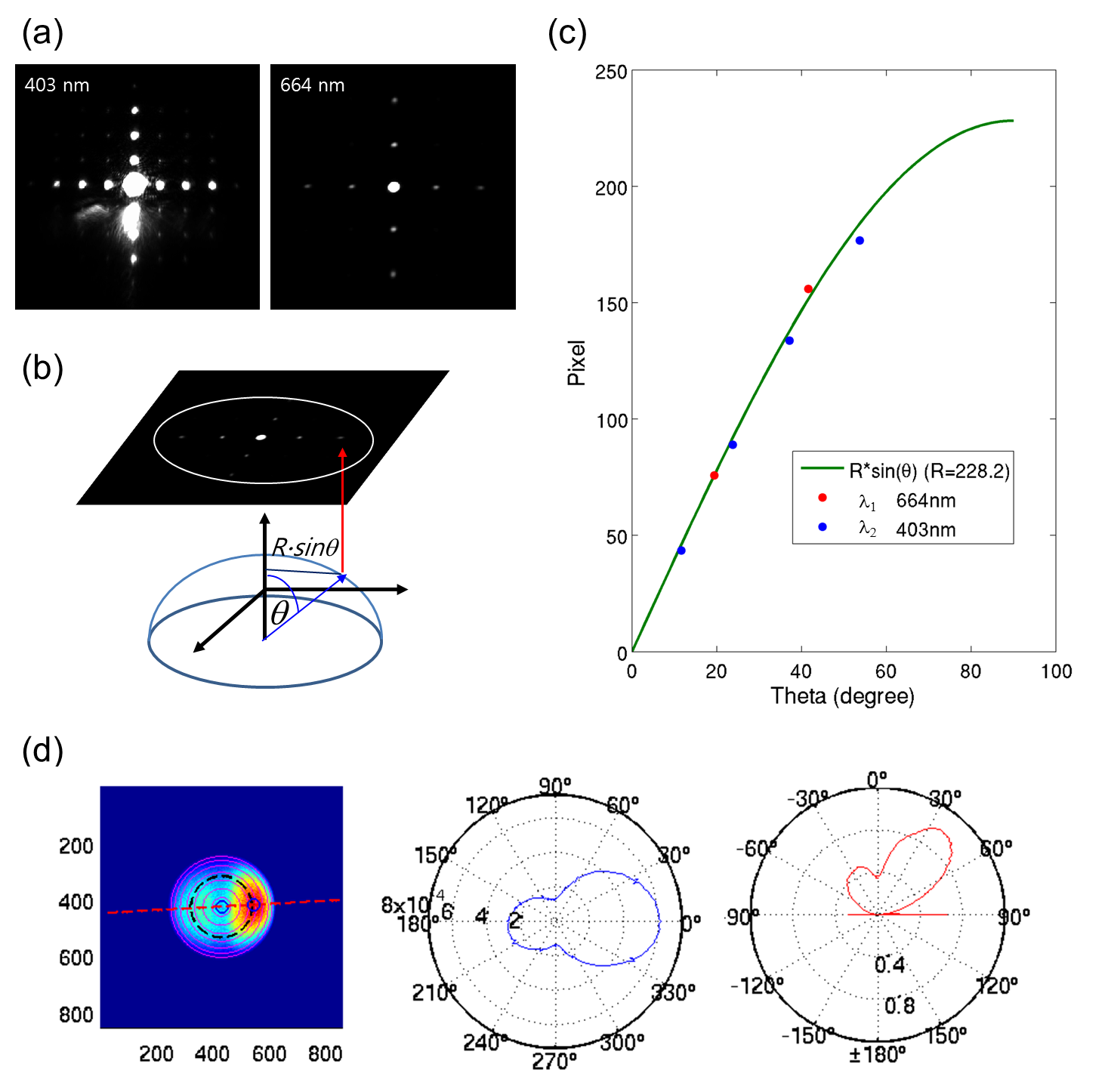
**

**Figure S4. Analysis of deflection angle** (a) 2D diffraction pattern of 2D grating with 2 μm spacing. The left (right) image was obtained using a laser with 664 nm (403 nm) wavelength. (b) Schematic of the relation between the 2D CCD image and hemisphere. (c) Graph of pixels and diffraction pattern obtained using 2D grating sample. (d) Analysis of measured Fourier-space image. Middle (right) image is a φ−plot (θ−plot) of the Fourier-space image.

**
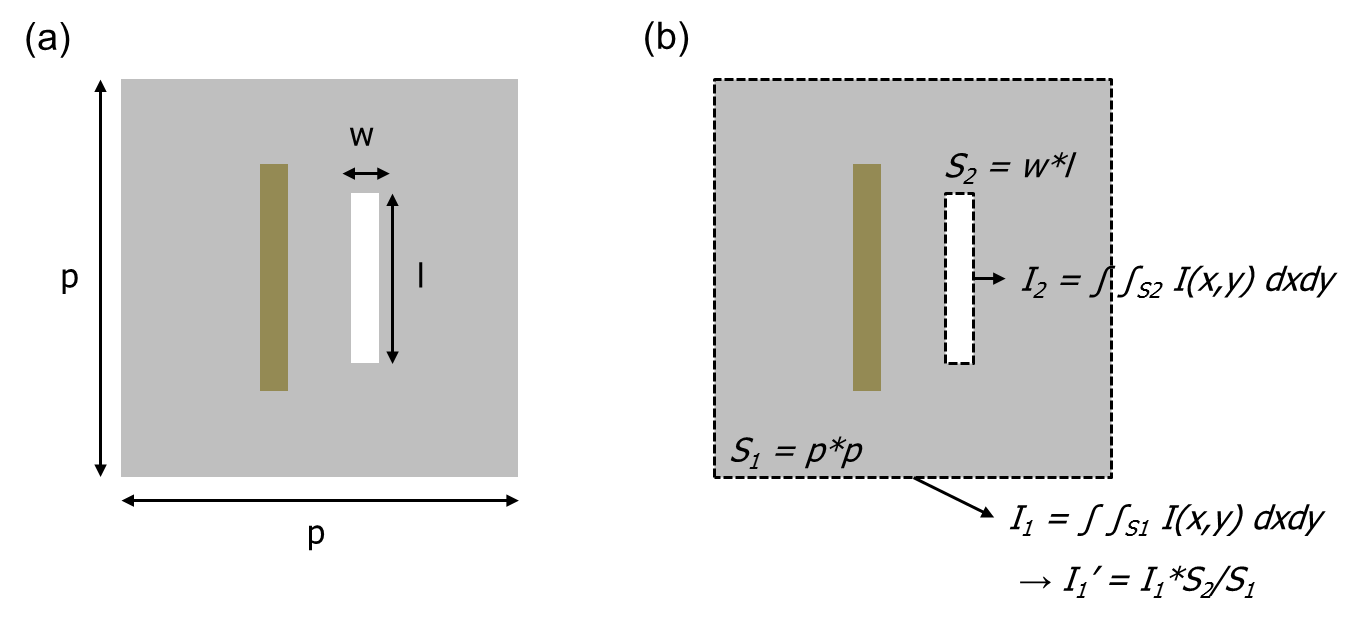
**

**Figure S5. Area and intensity of one slot-groove and one-lattice** (a) Schematics of dimensions of one slot-groove and one lattice. (b) Areas of one-slot and one-lattice. The *I_1_* and *I_2_* are the intensity measured through one-lattice and one-slot, respectively. The reference slot-intensity *I_1_’* corresponds to the *I_1_* multiplied by the area factor of *S_2_/S_1_*.

**
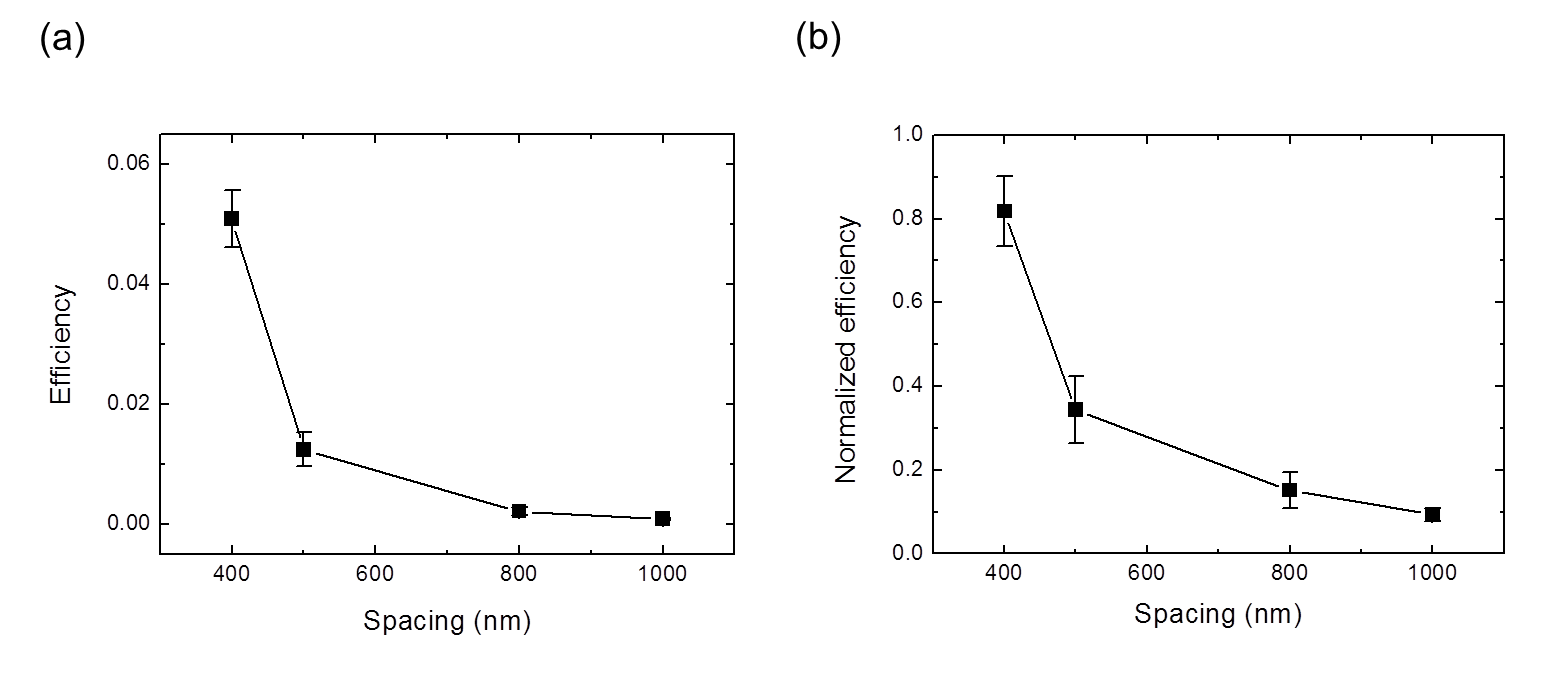
**

**Figure S6. Comparison of the emission efficiency** (a) Absolute unit-cell emission efficiency of slot-groove with different spacings (b) Hole-area-normalized emission efficiency of slot-groove with different spacings

**Table and Table Caption**

| Period | Absolute unit-cell  efficiency | Hole area-normalized  efficiency |
| --- | --- | --- |
| 400 nm | 0.04483 | 0.81820 |
| 500 nm | 0.01239 | 0.34404 |
| 800 nm | 0.00214 | 0.15187 |
| 1000 nm | 0.00083 | 0.09261 |

**Table S1.** An absolute unit-cell and a hole-area-normalized emission efficiency of slot-groove arrays with different spacings.
